# Responsible nudging for social good: new healthcare skills for AI-driven digital personal assistants

**DOI:** 10.1007/s11019-021-10062-z

**Published:** 2021-11-25

**Authors:** Marianna Capasso, Steven Umbrello

**Affiliations:** 1 Scuola Superiore Sant’Anna, Piazza Martiri della Libertà 33, 56127 Pisa, Italia; 2 Department of Values, Technology, & Innovation, School of Technology, Policy & Management, Delft University of Technology, Jaffalaan 5, 2628 BX Delft, The Netherlands

**Keywords:** Artificial intelligence, Nudging, Medical AI, Technoethics

## Abstract

Traditional medical practices and relationships are changing given the widespread adoption of AI-driven technologies across the various domains of health and healthcare. In many cases, these new technologies are not specific to the field of healthcare. Still, they are existent, ubiquitous, and commercially available systems upskilled to integrate these novel care practices. Given the widespread adoption, coupled with the dramatic changes in practices, new ethical and social issues emerge due to how these systems nudge users into making decisions and changing behaviours. This article discusses how these AI-driven systems pose particular ethical challenges with regards to nudging. To confront these issues, the value sensitive design (VSD) approach is adopted as a principled methodology that designers can adopt to design these systems to avoid harming and contribute to the social good. The AI for Social Good (AI4SG) factors are adopted as the norms constraining maleficence. In contrast, higher-order values specific to AI, such as those from the EU High-Level Expert Group on AI and the United Nations Sustainable Development Goals, are adopted as the values to be promoted as much as possible in design. The use case of Amazon Alexa's Healthcare Skills is used to illustrate this design approach. It provides an exemplar of how designers and engineers can begin to orientate their design programs of these technologies towards the social good.

## Introduction

Digital health provides clinicians, patients, caregivers, and care-receivers, generally construed, as well as the whole health system with new tools and possibilities, ranging from the use of wearables and connected medical devices such as smartwatches and activity trackers (Lu et al. 2019) to the spread of AI decision-making systems, such as chatbots, digital personal assistants or persuasive apps that can help in monitoring health metrics (Valtolina et al. 2020; Zhang and Wan 2019). The future potential for such systems in healthcare seems high due to the far-reaching implications that the diagnosis and prevention capabilities of AI-driven systems may have in integrating or even replacing more traditional medical practices and relationships.

Now, during the pandemic, AI is also helping to provide personalised information and recommendations for patients who have symptoms of COVID-19 (Buoy Health 2021). Specifically, AI-driven digital personal assistants—e.g., Google Assistant, Apple Siri, Amazon Alexa—are now used in a wide variety of scenarios—such as consumer markets, work, smart homes and others—and with advanced intelligence and interaction capabilities, they assist users in their tasks (Maedche et al. 2016). The novelty of such technologies is that they have also begun to prove their potential as digital health tools for monitoring, consulting, and providing tips and guidance, thus as intermediaries between the healthcare system as a whole and the public (Sezgin et al. 2020).

The scope of this paper is to analyse AI-driven digital personal assistants that are now upskilled with healthcare capabilities by looking at a particular case study, that is, Amazon Alexa's new Healthcare Skills. This will be done with the help of a multi-tiered value sensitive design (VSD) approach by evaluating a specific case of a new Amazon Alexa’s Healthcare Skill: i.e., the provision of digital health nudges—or recommended courses of action and suggestions—that are personalised to users. To do this, this paper is divided into the following sections. §2 explores the notion of nudging and its application in digital environments. §3 introduces a multi-tiered value sensitive design (VSD) approach as a means of designing AI-driven digital personal assistants that incorporate digital health nudges to not only avoid doing harm by operationalising the AI for Social Good (AI4SG) principles as norms but to actively promote social good. It does this by operationalising higher-order values such as the United Nations Sustainable Development Goals (SDGs) and the EU High-Level Expert Group on AI (HLEG AI) values. The case study of Amazon Alexa’s new Healthcare Skills is illustrated through the four-stage iterative process of a combined AI4SG-VSD approach, which are respectively: (1) context, (2) value identification, (3) formulating design requirements, and (4) prototyping. Finally, §4 provides some conclusions.

## Digital nudging in healthcare

The notion of nudging derives from the work of Thaler and Sunstein (2009), which advocates a liberal and paternalistic choice architecture. A nudge is described as "any aspect of the choice architecture that predictably alters people's behaviour without forbidding any options or significantly changing their economic incentive" (Thaler and Sunstein 2009, p. 6). Nudging uses behavioural sciences and economics principles to elicit beneficial behaviours from individuals, without undermining their deliberative choice and freedom. A nudge overcomes agents' cognitive defects, or lack of information and behavioural biases, and steers them towards target acts that are deemed to be good for them (Sunstein 2017).

The term "digital nudging" emerged only recently in engineering and computer systems literature and is defined as the "use of user-interface design elements to guide people's behaviour in digital choice environments" (Weinmann et al. 2016). There are several technological systems in healthcare that use and rely on the assumptions of nudging. Among these, there are apps that send notifications based on nudge design to prevent the progression of mild cognitive impairment in elderly patients (Pietrabissa et al. 2019). Other examples that fall more squarely in digital nudging in the healthcare context include AI-driven digital personal assistants to support diagnosis and monitoring. Such systems influence and manage users' behaviours and have a significant impact on both caregivers and care receivers, as well as their families.

Indeed, market-driven corporations such as Apple, Google or Amazon are now incorporating data-driven and personalised nudges in their products. AI-driven digital personal assistants are an example of such products. For example, users can be ‘nudged’ by Amazon’s Alexa, which can collect users’ data and preferences, shape different aspects of their choice environment and push towards desired results, thus having considerable power in affecting decision-making processes in a vast realm of contexts, from business and markets to other sensitive domains such as healthcare (Cai 2020).

However, little research has been conducted on whether digital nudges improve the efficacy of healthcare (Byambasuren et al. 2018). Conversely, a growing concern has been raised that apps and other AI medical tools may track, collect, and share data in opaque and potentially misaligned ways (Loria 2019).

Digital nudges follow the same principles and modalities of nudges as such. However, the fundamental difference between traditional nudges and digital nudges lies in the latter allowing for greater versatility and opportunities for choice architects due to the more dynamic, informational, and automated character of the digital environment (Meske et al. 2019). In addition, new digital patient-centric nudges, with tools such as simplifications, default settings, decision staging, feedback, reminders and others, offer virtual medical care and assistance in and outside hospitals and in domestic or commercial care practices even outside the healthcare domain (Meske et al. 2019).

Using Big Data and Predictive Analytics techniques, a digital nudge is ubiquitous, emergent, interconnected, and capable of continuously reconfiguring itself due to its feedback from its environment and interactions with users and other systems. As a matter of fact, Big Data nudges have been defined as a special kind of nudge: *hypernudges* due to their networked and dynamic nature (Yeung 2017).

Nudging is not merely a value-free and neutral tool: indeed, scholars have already recognised that nudges always contain value judgements and deal with a dominant understanding of societal values or norms that are considered morally or politically acceptable by choice architects (Prainsack 2020; McMahon 2015; Jones et al. 2013).

In the digital realm, tech companies or private actors may constitute choice-architects with an external, independent and, in some instances, unaligned values-metrics relative to those of their nudgees' goals and values. Indeed, in certain cases, digital nudges could steer the behaviour of human users away from what may benefit them, leading to a disparity between their actions and goals (Burr et al. , 2018). In particular, in the healthcare context, the introduction of new actors and new digital practices mediated by AI-driven systems can shape the responsibility, roles and credibility of both health professionals and patients, leading to a reconfiguration of the entire healthcare system (van Wynsberghe and Li 2019). Scholars have raised concern about the "Googlization of health” research that opened the way to tech companies such as Google, Amazon, and Facebook to collect, track, and store health data (Sharon 2016; 2021). Health data are not solely created by clinicians in electronic medical records but now extend to encompass data from fitness and health apps, behavioural data tracked in digital environments, data based on online interactions, and the contents amassed on social networks, among others.

Therefore, a question of crucial importance in collecting and using healthcare data is the nature and value of those data as inherently *public* data (c.f., Prainsack 2020). Whereas individual health data would (and should) continue to be treated as private (i.e., individual) health data, the data can also be read as public data, given that its collection and use in AI-driven systems have a public impact. Despite proponents of nudging tend to focus on individuals and their freedom of choice—since nudges are primarily described as individual interventions that help to promote more rational and healthy behaviours—the use of nudges may profoundly impact institutional and social structures (see Lepenies and Małecka 2015). On the extensive literature on nudging in healthcare, very little engage with social determinants of health and often mislead the public nature of nudges (MacKay and Quigley 2018).

Behavioural influences such as nudging techniques are always positioned in a more extensive system, where policymakers or private actors and governments layout values and norms, preferences, and political or social factors. The effects of digital nudges within the healthcare field are thus not merely visible on an individual level; they are equally effective and persistent on a collective level and need a justification on the values they underpin and promote.

Thus, it is essential to make explicable the values that are articulated by digital nudges – general social values or values tailored for the individual (c.f., Barton and Grüne-Yanoff 2015) or a specific group (stakeholder contextual values) – and align AI-driven systems in the field of healthcare to not only avoid doing harm (nonmaleficence) but to actively contribute to social good (beneficence). Specifically, in the healthcare domain, there is a need to identify and regulate the different sources of influences such as digital nudges in the broader social environment and to assess *ex-ante* the peculiarities and the relevant values inherent to the care practices that such influences are going to interfere with.

It is not only the public nature of digital nudges at stake, but the question about the public nature and value of health that should benefit society as a whole and prevent persons or groups of people from experiencing undue harm. In this sense, when engaging in a discourse on AI-driven systems that incorporate digital health nudges that should actively contribute to social good, one cannot disregard the question of what may count as a just healthcare system and the reconsideration of an individualistic approach to health.

The COVID-19 pandemic has shown the need to initiate a thorough discussion on health and healthcare's role in citizens’ lives. For example, the necessity of thinking public health as a social good has been in the recent efforts of the World Health Organization, which was engaged in providing countries all over the globe equitable access to vaccines (WHO 2020). Furthermore, in recent years, many scholars have tried to frame health in terms of a global public good, seeing in it not a private good but rather a concept that requires innovative collective action at the global level (Moon et al. 2017; Abdalla et al. 2020). Public goods are non-excludable and non-rival, meaning that no one is excluded from their consumption, and one person’s consumption does not prevent anyone else from benefiting them (Smith 2003). If we translate this discourse on health and healthcare’s role in citizens lives, the access to healthcare and treatments should not be arbitrarily limited but considered inherently public: i.e., no one is excluded from it and is not-rivalrous. The healthcare ecosystem is increasingly becoming an ‘e-health ecosystem’. This e-health ecosystem can be understood as a socio-technical system that is composed of an entanglement of technical, social, and institutional dimensions and stakeholders that in collaboration provide values and services (Nykänen 2017). For this reason, society as a whole is expected to create and maintain a healthcare ecosystem that can correctly evolve, approximate towards being a public good, and can assist individuals in the above sense. However, beyond such consideration on individual access to healthcare and treatments, health can also be understood as public, aimed to fit the characteristics of non-excludability and non-rivalry. Although there are many different conceptualisations of public health through the lens of the conception of public goods, these have also led to criticisms (Bernstein and Randall 2020). Still, the importance of the concept and practice of public health primarily depend on structural conditions and on the collective call for action to address and provide health-related public goods, public health programs, and policies (Giubilini and Savulescu 2019; Anomaly 2021).

In particular, the proposal of framing health as a *global* public good can be a means to include other sectors and regulators beyond the healthcare system itself in the design and justification of health policies, strategies and plans. The social determinants of health and their implications should be included in a new and more comprehensive approach to monitoring, implementing and evaluating health, understood not as the sum of individual goods of specific persons but as both the outcome and pre-condition of much broader socio-economic processes and practices. To ensure global access and benefit concerning health and healthcare, it would be effective to identify a rationale, methodologies, and means to account for the challenge of fair and democratic redistribution, as well as social justice.

A multi-tiered value sensitive design (VSD) approach that operationalises higher-order values such as the United Nations Sustainable Development Goals (SDGs) and the EU High-Level Expert Group on AI (HLEG AI) values can constitute a reasonable solution to the issue of strengthening global responsibility and governance to improve health. Higher-order values like those expressed by SDGs and those higher-level values specific to AI, like those described by HLEG AI, can serve as a good approximation of what we consider a collective action to individuate public goods to be promoted at the global level.

This approach has two aims. First, it aims to reconfigure the healthcare system by introducing new actors and indicators that determine and make explicit the values to be promoted as public and collectively beneficial. Second, it aims to identify norms and design requirements for AI-driven systems—in this specific case, AI-driven digital personal assistants—to better monitor and promote health equity and an inclusive, transparent and accountable development of socio-technical systems globally.

## Towards AI for social good

As already mentioned, digital nudging is a continually growing practice within the domain of health care, technology, and their intersection. However, given the potentially deleterious consequences of misaligned digital nudging, as well as its potential boons if employed responsibly, it makes sense that the responsible innovation of AI-driven digital personal assistants in the field of healthcare to be aligned with a design approach that is principled on similar commitments to avoid harm and actively contribute to doing good. Therefore, many scholars are now attempting to identify technical requirements for AI-driven systems to ensure that these technologies are designed to protect and promote relevant ethical and societal values in healthcare (Mittelstadt et al. 2016; London 2019; Vayena et al. 2018).

Floridi et al. (2020) provide what is arguably the most comprehensive set of norms for guiding designers of AI-driven systems to avoid (most) harms (Umbrello and van de Poel 2021). The seven factors—AI4SG norms—are a set of principles that are particularly relevant for the design of AI towards social good (Floridi et al. 2020). Table 1 lists the AI4SG factor along with a summative imperative that Floridi et alia state that designers must follow to put these factors into practice.

**Table 1:** AI for social good meaning and factors

AI4SG factor	AI4SG factor imperative
1. Falsifiability and incremental deployment	AI4SG designers should identify falsifiable requirements and test them in incremental steps from the lab to the “outside world” (Floridi et al. 2020, p. 7)
2. Safeguards against the manipulation of predictors	AI4SG designers should adopt safeguards that (i) ensure that non-causal indicators do not inappropriately skew interventions and (ii) limit, when appropriate, knowledge of how inputs affect outputs from AI4SG systems to prevent manipulation (Floridi et al. 2020, p. 8)
3. Receiver-contextualised intervention	AI4SG designers should build-decision-making systems in consultation with users interacting with and impacted by these systems; with understanding of users’ characteristics, of the methods of coordination, and the purposes and effects of an intervention, and with respect for users’ right to ignore or modify interventions (Floridi et al. 2020, p. 9)
4. Receiver-contextualised explanation and transparent purposes	AI4SG designers should choose a Level of Abstraction for AI explanation that fulfils the desired explanatory purpose and is appropriate to the system and the receivers; then deploy arguments that are rationally and suitably persuasive for the receivers to deliver the explanation and ensure that the goal (the system’s purpose) for which an AI4SG system is developed and deployed is knowable to receivers of its outputs by default (Floridi et al. 2020, p. 14)
5. Privacy protection and data subject consent	AI4SG designers should respect the threshold of consent established for the processing of datasets of personal data (Floridi et al. 2020, p. 16)
6. Situational fairness	AI4SG designers should remove from relevant datasets variables and proxies that are irrelevant to an outcome, except when their inclusion supports inclusivity, safety, or other ethical imperatives (Floridi et al. 2020, p. 18)
7. Human-friendly semanticisation	AI4SG designers should not hinder the ability for people to semanticise (that is, to give meaning to and make sense of) something (Floridi et al. 2020, p. 19)

**Fig. 1:**
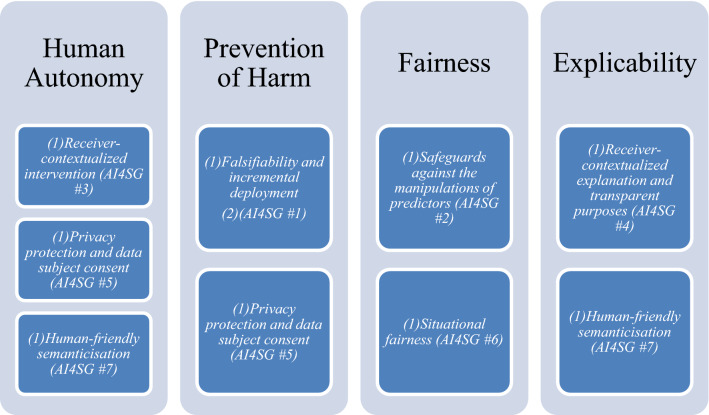
Relationship between higher-order values of the EU HLEG on AI and AI4SG norms. 2021 Source: Umbrello and van de Poel (2021)

The seven norms should not be read as rank-ordered, but mutually co-varying and co-constituting one another in design paradigms. Similarly, and more relevant to the specific types of technologies in question, they seamlessly map onto the higher-level, more abstract values of the EU High-Level Expert Group on Artificial intelligence (HLEG AI): *human autonomy*, *prevention of harm*, *fairnes*s and *explicability* (see Fig. 1) (High-Level Expert Group on AI 2019).

For the sake of space, this article does not discuss in depth the definitions or examples of the seven factors; Floridi et alia do so already at length (Floridi et al. 2020). However, what is important here is that the AI4SG factors function like *norms* as per van de Poel's (2013) characterisation of norms as being framed as 'maximising' or 'minimising' specific values or design requirements, thus bridging the gap between abstract values (e.g., HLEG AI, United Nations Sustainable Development Goals (SDGs) and concrete design requirements (van de Poel 2013). More clearly stated, the above shows that the norms via AI4SG factors provide a bridge between higher-level AI values like those of the HLEG AI and more technical design requirements. However, for this approach to be operationalised by designers, a principled design methodology is required to allow this bridging between abstract values and norms to be adopted systematically. For this reason, we adopt the value sensitive design (VSD) approach to technology design as the methodology of choice. VSD is a principled approach to technology design that seamlessly incorporates the 'values-norms-design requirements' structure as a foundational method (van de Poel 2013).

### Value sensitive design

As mentioned, this article aims to adopt and illustrate a multi-tiered VSD methodology to design AI-driven digital personal assistants that responsibly incorporate digital nudges within healthcare. Thus, the HLEG AI principles are understood as the more general values from which more specific values can be derived for *doing good*. At the same time, the normative AI4SG factors are used as the boundary conditions for *avoiding harm* (c.f., Umbrello and van de Poel 2021).

**Fig. 2:**
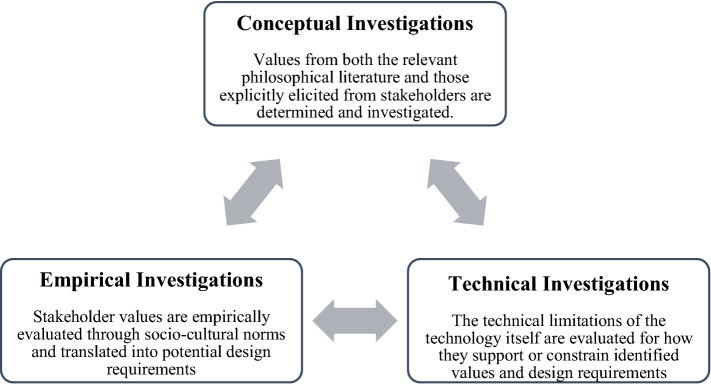
The recursive VSD tripartite framework employed in this study. Source, Umbrello (2020)

Currently, there are over two decades worth of scholarship directly on the VSD approach that explore its philosophical foundations (Winkler and Spiekermann 2018), methodological issues and capabilities (Le Dantec et al. 2009), as well as its potential applications to existing and future technologies (Umbrello and De Bellis 2018). Value sensitive design is often defined as "a theoretically grounded approach to the design of technology that accounts for human values in a principled and comprehensive manner throughout the design process" (Friedman et al. 2013, p. 2). The primary methodological objective of VSD is an explicit investigation and incorporation of moral values in design. It does this through the recursive feedback of three iterative stages or 'investigations': conceptual, empirical and technical, as shown in Fig. 2.

Conceptual investigations involve (1) identifying both direct and indirect stakeholders that are or will be affected by the system and (2) formulating working definitions and prima facie value tensions that may arise. Empirical investigations examine stakeholders' contexts and emerging values, eliciting their values and reformulating the working definitions of the conceptual investigations as necessary. Finally, technical investigations look at the discrete technology in question, determining how the architecture of the technology can support or constrain the values in question.

**Fig. 3:**
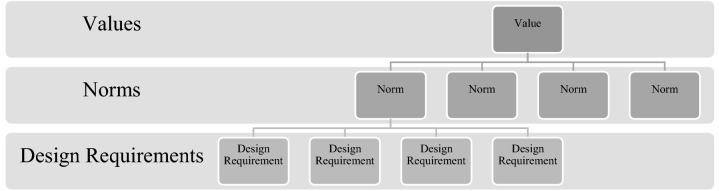
Values hierarchy. Source: van de Poel (2013)

Tools like the *values hierarchy* formulated by van de Poel (2013) are useful in helping designers to translate what are often abstract values into more tangible design requirements (see Fig. 3). A values hierarchy is fundamentally built on three primary layers: 1) values, which are often general and understood as needing to be promoted and designed for as much as possible; 2) norms, which are boundary conditions or prescriptions for action, and 3) design requirements, as specific technical requirements that should be designed for as much as possible.

### VSD for responsible nudging

**Fig. 4:**
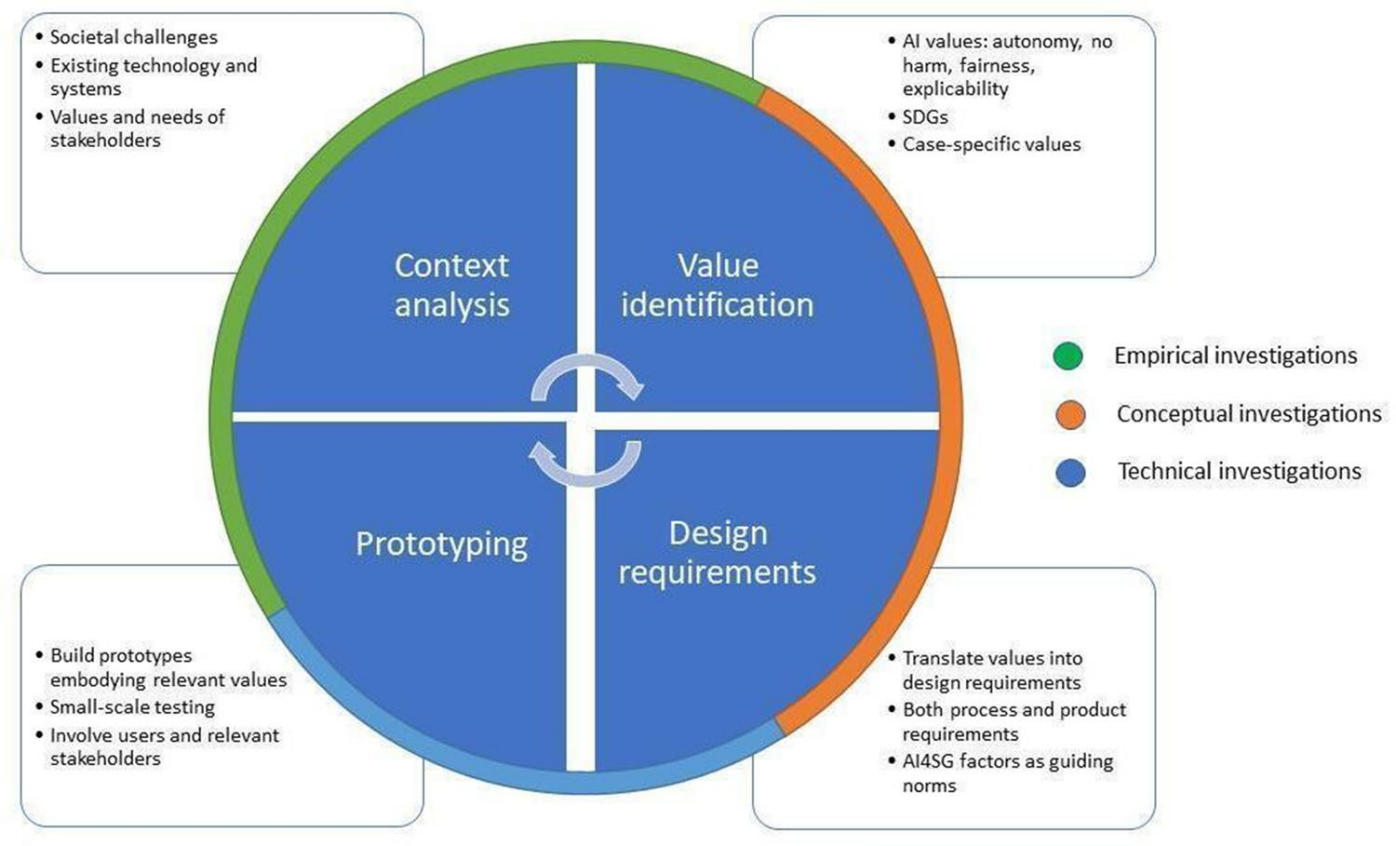
AI4SG-VSD design process. 2021 Source: Umbrello and van de Poel (2021)

To illustrate how the VSD approach can be used to design responsible nudging, we take up the example of Amazon's Alexa digital personal assistant as the use case. Likewise, given work that has already been done on adapting the VSD approach to AI-driven technologies, we adopt the general design program formulated by Umbrello and van de Poel (2020) as the starting point (Umbrello and van de Poel 2021). Figure 4 outlines how designers can begin their investigations in their design program. Albeit differing from one project to another, the proposed framework provides the general outline that practitioners can follow to ensure they touch on the fundamental points presented in this framework.

The four stages of the iterative process are: (1) context, (2) value identification, (3) formulating design requirements, and (4) prototyping. For the sake of space, we take up the approach directly and illustrate its application to the use case of Amazon's Alexa. In the last part of the article, we aim to discuss and explore the design of the Amazon Alexa Healthcare Skills prototype, albeit *ex post facto* in this case, using the framework described above (Fig. 4).

#### Context analysis

In 2019 Amazon announced a new partnership with the UK's National Health Service (NHS). This partnership enabled Amazon's digital personal assistant Alexa to offer NHS health advice to users at home (Department of Health and Social Care 2019). Moreover, in the new Alexa Healthcare Skills announced in collaboration with six health companies, Amazon says that now Alexa can follow the US Health Insurance Portability and Accountability Act of 1996 (HIPAA) and transmit and receive protected health information. These new Alexa Skills are designed to help users manage different healthcare needs such as the coordination and scheduling of appointments, care plans, healthcare account information, the tracking and monitoring of vitals and symptoms, and, finally, the receiving of insights and Health Nudges—or recommended courses of action and suggestions—that are personalised to them (Jiang 2019). Furthermore, during the COVID-19 pandemic, Amazon released additional Alexa features to help users stay at home, providing information and guidance about the virus (Amazon 2020).

The sociocultural contexts in which a technology is being developed is crucial to assess its design and deployment. For example, Amazon is not a healthcare company but can now handle health data and patient information. Likewise, Alexa is a general AI-driven digital personal assistant, commonly used for a wide variety of purposes, like playing music or giving information about the weather, among many other things. In the case of Alexa's new Healthcare Skills, the context of use, which can be understood as the motivating force beyond its development, includes a diverse range of factors: the need to reduce the pressure and burden on NHS, healthcare companies and clinicians, mainly by providing information on common illness, the need to render easily accessible and valuable health information and tools especially to vulnerable groups such as elderly and frail patients at home and in residential and nursing homes, differently-abled patients, or, generally, to those who cannot always get access to care or know how and when to get such access, the need to improve patients' adherence to their medicines (Beaney and Kalorai 2020), and for more accurate, preventive and personalised medicine and more beneficial health outcomes (Chan et al. 2019). As such, with the addition of healthcare skills, it might be argued that this blurs the line between different contexts, namely recreational contexts and those of healthcare. The introduction of AI-driven digital personal assistants can improve the effectiveness of health communication and monitoring. Still, concerns may arise during their use in practice, based on the need for a comprehensive infrastructure that helps the integration between different contexts and health systems and health organisations, the compliance to exchange health data and personal information, and so on.

#### Value identification

*Values that are to be promoted by the design* Ensuring that the AI4SG-VSD approach avoids harming and actively contributing to social good requires gearing the approach towards collective socially desirable ends. This type of explicit adaptation is currently lacking in the existent proposals for AI4SG. In a similar fashion to Umbrello and van de Poel (2020), we adopt an explicit gearing towards the SDGs as the best estimation of collectively beneficial societal ends given that the UN developed them to favour collective action for all countries (UN Task Team on the Post 2015 Agenda 2013). Amazon's Alexa new Healthcare Skills design can be said to be part of an extensive network to support UN SDG #3, *Ensuring healthy lives and promoting well-being at all ages*. In particular, it may encourage SDG target 3.8: the achievement of universal health coverage, including financial risk protection, access to quality essential healthcare services and access to safe, effective, quality and affordable essential medicines and vaccines for all (United Nations n.d.).

*Values that should be respected, in particular, those values that have been identified concerning AI: respect for human autonomy, prevention of harm (nonmaleficence), fairness and explicability* This second level of values are values that are to be promoted, especially concerning AI. First, a classic tenet of bioethics is the principle of autonomy, understood as the right that individuals have to make free and meaningful decisions about their treatments. With AI, this principle is blurred, according to Floridi et al. (2018) because it implies a balance between the decision-making power that individuals retain for themselves and what they decide to delegate to systems such as AI (Floridi et al. 2018). The access to health data sets and the provision of medical advice and information are no longer restricted to the dynamics of the healthcare system nor the reciprocal relationship between clinicians and patients. Indeed, big tech corporations such as Amazon can answer and suggest health-related questions and behaviours. AI-driven systems can store, archive, collect, and analyse data in new and unprecedented modalities, which we already defined as *hypernudges*, raising concerns about privacy and individual autonomy. Without explicit informed consent, Amazon can monitor users' routine to improve its service (Cuthbertson 2019) and thus has the potential capacity to utilise and share data even for commercial and marketing purposes not related to health issues. The involvement of such private actors and automated systems in healthcare can thus entail a loss of control on personal data and a downplaying of care receivers' meaningful choices concerning treatments and assistance.

In this scenario, the emphasis on the value of *human autonomy* can help address the issue of data management on the part of external providers and explore the role that digital nudges may have in the decision-making processes and cognitive capabilities of their nudgees. For example, a pilot study in Staffordshire has explored the potential of Amazon Alexa for patients with diabetes or with other health and dependence needs such as anxiety and depression. The study has reported that Alexa can have hugely positive effects for both patients and family carers, such as an increased sense of independence and management of long-term conditions (Chambers and Beaney 2020).

*Nonmaleficence* involves understanding systems' capabilities and limitations to avoid possible harms caused by overusing or misusing AI technologies (Floridi et al. 2018). In the case of Amazon Alexa, privacy concerns are just one of the possible harms. Strictly related to the case under examination, we may also include other risks such as the unnecessary appointments and concerns on the part of patients, the possible disappearance of certain medical and caregiver professions, the reconfiguration of expertise and responsibilities in and outside the healthcare system, the interconnection of different domains and different values-metrics, and the predominant and potential worrisome impact that a market-driven system such as a private tech corporation may have on shaping healthcare agendas and research (see Sharon 2016; 2021).

The value of *fairness* aims to eliminate unfair discrimination and ensure that the use of AI creates shared benefits (or at least sharable) and avoids further harms, such as the undermining of existing social structures (Floridi et al. 2018). Amazon Alexa or similar digital personal assistants may exacerbate existing (digital) health divides between those who can afford them and those who cannot (Stokes-Lampard 2019). Moreover, there is no safeguard to prevent the possibility for third parties or hackers and malicious actors to steal data. Beyond the individual level of the nudgee and their right to privacy and healthcare, what is at stake is the collective dimension of justification on the means and modalities of possible interventions in healthcare. Therefore, there is reason to frame the issue of digital nudging also in terms of its socio-political relevance, with the aim to regulate and promote relational and equal forms of accessibility to digital health data and democratic—as open to discussion and contestation—modalities of control on those latter.

Finally, the value of *explicability* implies a need to understand AI-driven systems and thus make them intelligible and not opaque. There is also a need to hold to account the decision-making processes of AI, finding at least one agent that can be considered accountable for how the system works (Floridi et al. 2018). This is a complex issue due to the vast number of people and organisations that deploy and develop such technologies and to the fact that AI working is often difficult to understand and interpret by those agents.

In cases that involve AI, predictive analytics, profiling and hypernudges, generally understood, traditional informed consent or the manifest and transparent character of the publicity principle are often not sufficient elements to preserve and respect users' deliberative choices and freedom (Yeung 2015; Sunstein 2017). Beyond the individual level of users' behaviours, what is at stake in the use of digital health nudging is the nature and public value of health data and the social, economic and political consequences and factors this may entail, as some scholars have recently suggested (Prainsack 2020). And beyond the issues of transparency and publicity, we should assess and promote modalities that make the adoption of AI-driven systems and technological influences-types such as digital nudges also explainable by public agencies and stakeholders to citizens (Pasquale 2015; Santoni de Sio and Mecacci 2021).

*Context-specific values that are not covered by (1) and (2) and derive from the analysis of the specific context in phase, particularly values held by stakeholders* The last class of values is related to stakeholders' values and preferences. In the healthcare domain, the nature of care activity is conceived as a response to the needs of the 'other' or care receiver to determine the values to be included in systems design (see van Wynsberghe 2016). Digital personal assistants such as Alexa may mediate and radically change the patient-provider relationship and the specific care practice that the latter aims to enforce. For example, as a virtual clinician at home, Amazon Alexa can be used to overcome loneliness and isolation and provide social support (Chambers and Beaney 2020). Or, more broadly, digital personal assistants can affect the users' willingness to disclose aspects of personal and clinical life in various ways compared to the more traditional interaction that a patient may have with human caregivers (Debajyoti et al. 2020). Therefore, the value of social companionship—and, consequently, considering technological systems as social agents or entities—as a context-specific value should be put in a sharper focus. According to the recent work of Friedman et al. (2019), in the possible VSD tools, which are appropriated from the social sciences and can be used to investigate and reconstruct stakeholders' experiences, are included the use of values scenarios or sketches, or value-oriented semi-structured interviews or even the deployment of models for informed consent online (Friedman and Hendry 2019). These may be appropriate methods for identifying new emerging values or validating design solutions that can consider different descriptions and understandings of care practices and the different usage patterns and perceptions across age groups (Oh et al. 2020).

#### Design requirements

**Fig. 5:**
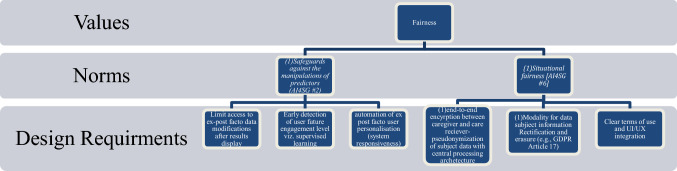
Translating the value of *Fairness* to design requirements through AI4SG norms

Among the host of VSD methodologies available for value analysis, a values hierarchy (Fig. 3) is particularly apt at visualising how higher-level values can be translated through norms and into design requirements. Naturally, the hierarchy can function both top-down (values → norms → design requirements) as well as bottom-up (design requirements → norms→ values) depending on the specifics of the design programs in which it is used. Figure 5 is an example of how a higher-level value can be translated through two of the AI4SG norms that are most relevant to it (c.f., Fig. 1) and into more tangible design requirements.

In Fig. 5, the value of *Fairness* is chosen as the exemplar of how to illustrate the usefulness of a values hierarchy as a tool for translating abstract values into concrete design requirements. *As* one of the higher-level values of the EU HLEG, *fairnes*s here can be translated through *at least* two AI4SG norms (2 and 6), illustrated in Fig. 1, and into some example design requirements that align with this value and its associated norms. Naturally, this is one of a host of examples of how this translation can be done. Of course, the design requirements or even values (depending on the direction of the hierarchy being undertaken) changes as a function of the various contextual factors of any given design domain. Simply put, the illustration is not an exhaustive exercise but rather 'opens up' the design space for multiple ways that designers can reach common goals. *Receiver-contextualised interventio*n (AI4SG #3) and *Receiver-contextualised explanation and transparent purposes* (AI4SG #4) overlap into the value of *Fairness* also, given that it can come into delicate tension with the requirements on the limits of transparency as such for the *safeguard against the manipulation of predictors* (AI4SG #2). This is to say that both the values and the norms co-vary and co-constitute one another and, to reiterate, are not rank-ordered but actually operationalise each other. For this reason, such exercises like this allow engineers to more clearly delimit the possible requirements needed for a salient design aligned with the norms characteristic of AI4SG and the more abstract values that are often difficult to conceptualise as concrete design requirements.

### Prototyping

The prototyping stage does not merely involve testing the technical aspects and functioning of technological systems but also, in a more fundamental way, the analysis of the ethical and social effects that can emerge from their deployment and field of use. This should include the development and co-creation of mock-ups, prototypes or field deployments that aims to identify value tensions and other factors and implications for the direct and indirect stakeholders and the technology at stake (Friedman and Hendry 2019). The case of Amazon Alexa is relevant, given its ubiquity, accessibility, and ease of adoption and implementation into existing health domains, in both caregivers and receivers. Alexa's pervasiveness exacerbates the technology's systemic interactions that follow from its widespread adoption. Technologies, like Alexa, can become pervasive across multiple vectors such as those of geography, culture, and demographics, among other factors (Friedman et al. 2017). At this stage, given its limited deployment, both technical and social/ethical functioning according to the guidelines can be pen tested securely. Emergent issues or misalignment can then result in the triggering of another iteration of this four-stage cycle.

## Conclusions

Digitisation of medicine brings with it a host of boons such as increased efficiency and accessibility. However, with these benefits may also emerge public concerns due to the specific capabilities of AI-driven systems and the possible sources of AI-influences on stakeholders and environments. This article proposes that AI-driven digital personal assistants that incorporate digital health nudging can be designed to avoid doing harm and promote social good. To do this, we suggest that the AI4SG norms form strong normative guidelines that designers can adopt to prevent (most) harms. Similarly, we show how these norms help translate more abstract values such as those of the HLEG AI and SDGs into tangible design requirements, thus actively promoting social good (as much as possible). Finally, the VSD approach is adopted as the general design methodology to encompass this multi-tiered strategy. If successful, the VSD approach provides, at least, a strong starting point for engineers and designers to design AI-driven systems that incorporates digital health nudges *for* human values and thus not only ameliorating potentially misaligned behaviour but actually contributing to the social good.

## Data Availability

Not applicable.
